# Usefulness of cancer-testis antigens as biomarkers for the diagnosis and treatment of hepatocellular carcinoma

**DOI:** 10.1186/1479-5876-5-3

**Published:** 2007-01-23

**Authors:** Fabio Grizzi, Barbara Franceschini, Cody Hamrick, Eldo E Frezza, Everardo Cobos, Maurizio Chiriva-Internati

**Affiliations:** 1 Laboratories of Quantitative Medicine, Istituto Clinico Humanitas IRCCS, Via Manzoni 56, 20089 Rozzano, Milan, Italy; 2 Department of Microbiology & Immunology, Texas Tech University Health Science Center and Southwest Cancer Treatment and Research Center, 3601 4th St., 79430 Lubbock, Texas, USA; 3 Department of Surgery, Texas Tech University Health Science Center and Southwest Cancer Treatment and Research Center, 3601 4th St., 79430 Lubbock, Texas, USA; 4 Department of Hematology & Oncology, Texas Tech University Health Science Center and Southwest Cancer Treatment and Research Center, 3601 4th St., 79430 Lubbock, Texas, USA

## Abstract

Despite advances in our cellular and molecular knowledge, hepatocellular carcinoma (HCC) remains one of the major public health problems throughout the world. It is now known to be highly heterogeneous: it encompasses various pathological entities and a wide range of clinical behaviors, and is underpinned by a complex array of gene alterations that affect supra-molecular processes.

Four families of HCC tumour markers have been recently proposed: *a) *onco-fetal and glycoprotein antigens; *b) *enzymes and iso-enzymes; *c) *cytokines and *d) *genes. A category of tumour-associated antigens called cancer-testis (CT) antigens has been identified and their encoding genes have been extensively investigated. CT antigens are expressed in a limited number of normal tissues as well as in malignant tumors of unrelated histological origin, including the liver. Given that cancers are being recognized as increasingly complex, we here review the role of CT antigens as liver tumour biomarkers and their validation process, and discuss why they may improve the effectiveness of screening HCC patients and help in determining the risk of developing HCC.

## Background

Hepatocellular carcinoma (HCC) is a common malignancy affecting approximately one million of people around the world every year and represents the fifth most common cancer worldwide with an incidence equal to the death rate [1-3]. The incidence of HCC is low in the occidental world and high in Southeast Asia and sub-Saharan Africa, however it has been rising in the last two decades in Europe, United states and Japan [3,4]. Furthermore, it is the second common cause of death in China, where its mortality rate is 20.37/100 thousand [5].

HCC primarily affects old people, reaching its highest prevalence among those aged 65 to 69 years old. Chronic infection by the hepatitis B virus is the most common cause of this neoplasia. Other important causes are cirrhosis, chronic viral hepatitis (hepatitis C virus, and hepatitis B plus D viruses), alcohol abuse, obesity, hemochromatosis, α1-antitripsin deficiency, and toxins similar to aflatoxin [1,3]. Accordingly, the major clinical risk factor for the development of HCC is liver cirrhosis since 70–90% of HCCs develop into a cirrhotic liver. Most HCCs occur after many years of chronic hepatitis that provides the mitogenic and mutagenic environments to precipitate random genetic alterations resulting in the malignant transformation of hepatocytes and HCC development [6]. In most cases, HCC is asymptomatic and has a low life expectancy.

Despite its significance, there is only an elemental understanding of the molecular, cellular and environmental mechanisms that drive disease pathogenesis, and there are only limited therapeutic options, many with negligible clinical benefit [7].

As for most types of cancer, hepatocarcinogenesis is a multi-state process involving different genetic alterations that ultimately lead to the malignant transformation of hepatocytes. HCCs are phenotypically (morphology and microscopy) and genetically heterogeneous tumors, possibly reflecting in part the heterogeneity of etiologic factors implicated in HCC development. Malignant transformation of hepatocytes may occur regardless of the etiologic agent through a pathway of increased liver cell turnover, induced by chronic liver injury and regeneration in a context of inflammation, immune response, and oxidative DNA damage. This may result in genetic alterations that affect supra-molecular processes [6].

The multi-state process of hepatocarcinogenesis is complex and heterogeneous and depends on a combination of many genetic, viral, and environmental factors. Different genes have been implicated in hepatocarcinogenesis including genes regulating DNA damage responses, genes involved in cell cycle control, genes involved in growth inhibition and apoptosis, and genes responsible for cell-cell interaction and signal transduction. The major molecular features of human HCC include aneuploidy and chromosomal aberrations, activation of proto-oncogenes, and inactivation of tumor suppressor genes [6-9].

However, this database has been obtained largely in a shatter and uncoordinated manner, typically in investigations in which aberrations in single genes have been analyzed in a group of HCC from humans and experimental animals. Although these data all seems to be helpful, they have not led to a coherent understanding of the complex mechanisms underlying HCC development, or to the identification of critical genomic and/or molecular aberrations that improve the carefulness of diagnosis or therapeutic interventions.

HCC is a non-linear complex disease that emerges from multiple spontaneous and/or inherited mutations that induce dramatic changes in expression patterns of genes and proteins that function in networks controlling critical cellular events [10-16]. Therefore, it is compulsory to identify HCC and the recurrence at its earlier period. A number of serum markers have been proposed and currently used as an effective method for detecting HCC for a long time [17]. Four families of HCC tumour markers have mainly been proposed and currently applied in the clinical practice [17]. In addition, a category of tumour-associated antigens called cancer-testis (CT) antigens has recently been identified and their encoding genes have been extensively investigated. CT antigens are expressed in a limited number of normal tissues as well as in malignant tumors of unrelated histological origin.

Given that cancers are being recognized as increasingly complex, we here review the role of CT antigens as liver tumour biomarkers and their validation process, and discuss why they may improve the effectiveness of screening HCC patients and help in determining the risk of developing HCC.

## HCC biomarkers: from biochemistry to genomics

Four distinct families of HCC tumour markers have been proposed and currently applied in the clinical practice: *(a) *onco-fetal and glycoprotein antigens; *(b) *enzymes and iso-enzymes; *(c) *cytokines and *(d) *genes [17]. However, it is compulsory to underline that a truthful specific biomarker for early detection, diagnosis or clinical outcome/prognosis of HCC is still undiscovered.

### Onco-fetal antigens and glycoprotein antigens

a) *Alpha fetoprotein (AFP) *is a fetal specific glycoprotein produced primarily by the fetal liver, yolk sac and gut, in the first trimester of pregnancy [18-20]. In normal conditions, its serum concentration falls rapidly after birth and its synthesis in adult life is repressed. Pathological elevation of AFP is seen in hepatocyte regeneration, hepatocarcinogenesis and embryonic carcinomas. AFP was first described as a marker for HCC by Abelev in the 1960s [21] and used as a serum marker for HCC in humans for many years. It has a sensitivity of 39%–65%, a specificity of 76%–94%, and a positive predictive value of 9%–50% [22]. However, greater than 70% of HCC patients have a high serum concentration of AFP because of the tumor excretion. HCC patients with a high AFP concentration (≥ 400 ng/mL) tend to have greater tumor size, bilobar involvement, massive or diffuse types, portal vein thrombosis, and a lower median survival rate. Though the measurement of AFP serves as an important tool in screening HCC patients, some reports have indicated that it has limited utility of differentiating HCC from benign hepatic disorders for its high false-positive and false-negative rates, and patients with acute exacerbation of viral hepatitis but no HCC may also have markedly increased AFP levels [17,23].

b) *Glypican-3 (GPC3) *is one of the members of heparan sulfate proteoglycans. Although it is highly expressed during embryogenesis and is involved in organogenesis, its exact biological function remains unknown. It binds to the cell membrane through the glycosyl-phosphatidylinositol anchors. GPC3 is able to interact with various growth factors that either stimulates or inhibits the growth activity. [17,23-25]. GPC3 mRNA and protein are expressed in >80% of human HCC but not in normal tissues except for placenta and fetal liver [26]. Recently, Nakatsura and Nishimura described GPC3 as a novel tumor marker for human HCC [27]. It was found in sera from 40–50% of HCC patients, although it was not detected in sera from patients with liver cirrhosis or chronic hepatitis, or in sera from healthy individuals [27].

### Enzymes and iso-enzymes

a) *Gamma-glutamyl transferase (GGTII) *is a glycosylated membrane enzyme, catalyses the transfer of Q-glutamyl groups from peptide donors such as glutathione to a variety of acceptors including dipeptides, free amino acids or water. GGT activity is modulated in many physiological and pathological conditions, including differentiation and carcinogenesis [28]. In the liver GGT is mainly secreted in serum by macrophagic Kupffer cells and endothelial cells of bile ducts and its activity increases in tissues with HCC [17]. Sensitivities of GGTII have been reported to be 74.0% in detecting HCC and 43.8% in detecting small HCC [29]. Recently, it has been shown that changes in GGTII occurring with changes in alcohol consumption were similar regardless of ethnicity.

b) *Alpha-l-fucosidase (AFU) *is a glycosidase primarily found in lysosome and involved in the degradation of a variety of fucose-containing fucoglycoconjugates [30]. The alterations of AFU catalytic activity in human cells, tissues and body fluids have a diagnostic value for human tumors including primary HCC [17,31-33], colorectal cancer [34] and ovarian cancer [35].The deficiency of AFU activity in female sera is probably a hereditary condition related to higher risk of ovarian cancer [30]. The persistently elevated AFU level in sera of patients with liver cirrhosis contributes to early detection of HCC [17,30].

c) *Des-gamma-carboxyprothrombin (DCP) *is an abnormal prothrombin protein that is increased in the serum of patients with HCC. Generation of DCP is thought to be a result of an acquired defect in the post-translational carboxylation of the prothrombin precursor in malignant cells. The reduction of gamma-carboxylase activity had been determined to be due to defective gene expression in HCC. In 8 large case-controlled studies, serum DCP was found to have a sensitivity of 48% to 62%, a specificity of 81% to 98%, and a diagnostic accuracy of 59% to 84% in differentiating patients with HCC from those with cirrhosis [36]. DCP is a well recognized tumor marker for HCC. It is an abnormal product from liver carboxylation disturbance during the formation of thrombogen, and acts as an autologous mitogen for HCC cell lines [17,37]. DCP was more sensitive and specific than AFP for differentiating HCC from nonmalignant chronic liver disease [38].

### Cytokines

a) *Vascular endothelial growth factor (VEGF)*, is an endothelial cell mitogen that induces and promotes angiogenesis and endothelial cell proliferation, which plays an important role in regulating angiogenesis, and which was initially identified as a vascular permeability factor. VEGF expression plays an important role in the development of HCC. Its degree of expression has been reported to be associated with tumor size and histological grade [17,38-42].

b) *Interleukin-8 (IL-8) *is a multifunctional CXC chemokine that affects human neutrophil functions, including chemotaxis, enzyme release, and expression of surface adhesion molecules [17]. IL-8 seems to have also direct effects on tumor and vascular endothelial cell proliferation, angiogenesis, and tumor migration. Recently, Kubo *et al *have shown that the expression of IL-8 in human HCC has more relevance to metastatic potential, such as vessel invasion, than to angiogenesis or cell proliferation [43]. Moreover, significant correlations of serum IL-8 levels with tumor size and tumor stage suggest that IL-8 may be directly or indirectly involved in the progression of HCC. These findings indicate that serum IL-8 may be a useful biological marker of tumor invasiveness and an independent prognostic factor for patients with HCC [44,45].

c) *Transforming growth factor-beta 1 (TGF-β1) *is a multifunctional cytokine involved in the regulation of growth and differentiation of both normal and transformed cells. TGF-*β*1 is a negative growth factor which correlates with cellular immunosuppression during the progression of HCC. It has been reported that TGF-*β*1 mRNA and its protein were over-expressed in HCC tissues, and plasma TGF-*β*1 was elevated in patients with HCC. Its serum level in HCC patients has been shown to be elevated compared with those in healthy adults and patients with non-malignant hepatopathy [17,46]. TGF-*β*1 mRNA was over-expressed in HCC compared with surrounding liver tissues, especially in small-sized and well-differentiated HCCs. In the liver, endogenous factors, such as TGF-*β*1 may be involved in induction of apoptosis. In HCC, birth and death rates of cells are several times higher than in surrounding liver. Consequently, tumor promoters may act as survival factors, *i.e*., inhibit apoptosis preferentially in precancerous and even in malignant liver cells, thereby stimulating selective growth of precancerous lesions [47].

d) *Tumor-specific growth factor (TSGF) *is a product synthesized and released by malignant cells. It results in blood capillary amplification surrounding the tumor, into peripheral blood during its growing period. Thus, the higher presence in serum of TSGF can be used as diagnostic marker in detecting HCC. Its sensitivity can reach 82% at the cut-off of 62 UI/mL [17,48]. TSGF is the first tumor biomarker which obtained the approval by Chinese government to land the market [48].

It is essential to emphasize that the quantification of cytokines in serum/plasma, collected prior to the clinical recognition of HCC, may provide insights into the nature of a host immune system environment that contributes to cancer development and/or growth. Such studies need to be designed with knowledge of the biology of the cytokines to be measured, and of the limitations inherent in the measurement of these molecules. Also, many cytokines are labile and/or are produced locally in immune system organs and not distributed systemically, so they cannot be detected in serum/plasma.

### Genes

a) *Alpha-fetoprotein mRNA (AFP mRNA) *It has recently been demonstrated that AFP mRNA expression detected by reverse-transcription polymerase chain reaction (RT-PCR) in peripheral blood 1 week after surgery correlated with the recurrence of HCC and was a good predictor for tumor recurrence [49]. In 1994, Matsumura *et al*. first reported that single HCC cell in circulation could be detected by means of RT-PCR, targeting AFP mRNA [50]. Subsequently, many investigators reported the value of AFP mRNA as a predictor for HCC recurrence, but the results are rather controversial. This may be due to the blood-borne dispersion of both tumor cells and normal liver cells during surgical manipulation and the mis-transcription of mRNA encoding AFP by peripheral mononuclear cells.

b) *Gamma-glutamyl transferase mRNA (GGT mRNA) *can be detected in the serum and liver tissues of healthy adults or patients with HCC, non-malignant hepatopathy, hepatic benign tumor, and secondary tumor of the liver [17]. The genetic regulation of GGT is complex. The human enzyme is encoded by a multi-gene family that includes at least seven genes, but only one (gene I) appears to code for an active enzyme. These mRNA products are named after the organs or cells from which they were first characterized: mRNA firstly described in fetal liver *(type A)*, the mRNA isolated from the human hepatoma cell line HepG2 cells *(type B) *and the mRNA isolated from human placenta *(type C) *[28]. Type A is predominantly expressed in normal liver tissues or liver tissues without malignant hepatopathy, benign tumor or secondary liver cancer. On the contrary type B is commonly found in cancerous tissues of HCC. Sheen *et al*. have demonstrated that patients of HCC with type B gamma-GTP mRNA both in cancer and in non-cancerous tissue had a worse outcome, earlier recurrence, and more post-recurrence death [51]. The authors suggested that HepG2 mRNA might be of diagnostic value [52].

c) *Human telomerase reverse transcriptase mRNA (hTERT mRNA) *has been shown to be a rate-limiting determinant of the enzymatic activity of human telomerase. Human telomerase is a ribonucleoprotein that has three major components: human telomerase RNA component (hTERC); human telomerase-associated protein 1 (hTEP1); and human telomerase reverse transcriptase (hTERT). Of the three components, hTERT is the catalytic subunit responsible for telomerase activity. Telomerase is expressed in most human cancers and immortal cell lines but is inactive in normal somatic cell lines or tissue. Recent reports support the concept that activation of telomerase may be an important and obligate step in the development of most malignant tumors, including human HCC [53]. Telomerase activity is closely correlated with hTERT expression. A weak expression of hTERT and telomerase activity was observed in 30% of non-cancerous tissues, while strong hTERT expression and enzyme activity were observed only in HCC. The cellular origin of hTERT expression in non-cancerous liver is still controversial, because infiltrating lymphocytes, precancerous liver parenchymal cells, and harboring micro-metastases of cancer cells may be responsible. This ambiguity makes the value of hTERT transcripts in the diagnosis of HCC and the feasibility of the promoter in gene therapy uncertain [54-56].

d) *Insulin-like growth factor II-mRNA (IGF-II) *Recently, it has been shown that the abnormal expressions of IGF-II mRNA might be a useful tumor marker for HCC diagnosis, differentiation of extra-hepatic metastasis and monitoring postoperative recurrence. Moreover, serum IGF-II level seems to be an independent serologic marker or a complementary tumor marker to AFP for diagnosis of small HCC [57].

It can be stressed that several other biomarkers have been proposed and preliminarily used in the early detection of HCC. AFP and DCP are, however, the most useful serum tumor markers for detection of HCC, and the simultaneous determination of these markers might improve the accuracy, especially in differentiating HCC from precancerous lesions, and nonmalignant hepatopathy.

A family of *tumour-associated antigens *(TAAs) has recently been identified and their encoding genes have been extensively investigated. Among them CT antigens are expressed in a limited number of normal tissues as well as in malignant tumors of unrelated histological origin (including HCCs), and are being vigorously pursued as targets for new therapeutic strategies. They are also being evaluated because of their role in oncogenesis and the recapitulation of portions of the germ line gene-expression program, which might contribute characteristic features to the neoplastic phenotype such as immortality, invasiveness, immune evasion, hypomethylation and metastatic capacity.

## The discovery of tumour-associated antigens

One of the most controversial issues in immunology for over a century has been whether an effective immune response can be elicited against malignant tumors [58]. Based on recent immunological developments, there is no doubt that the immune system can recognize and eliminate malignant cells. In 1967 Frank Macfarlane Burnet introduced a general hypothesis suggesting that lymphocytes are continually patrolling tissues and eliminating transformed cells, presumably via recognition of TAAs, a process he called "immunosurveillance" [59]. It is known today that the immune system is capable of discriminating between benign and malignant cells based on specific recognition of aberrantly expressed proteins or peptide fragments in the context of major histocompatibility complex (MHC).

Thus, the proteins encoded by abnormally expressed genes of a tumour cell may represent potential targets for immune cells, serving as tumour-associated rejection or regression antigens. With the exception of melanoma, few candidate TAAs have been described for human malignancies [60,61].

The introduction of a genetic approach of T-cell epitope cloning and subsequently an innovative method through the *serological analysis of recombinant tumor cDNA expression libraries with autologous serum *(SEREX) has greatly expanded the identification of a promising class of TAAs called CT antigens. Many TAAs have been identified using a genetic approach in which a cDNA library from tumour cell mRNA is generated in a eukaryotic expression system. Bacterial generated pools of cDNA, containing between 50–200 individual clones, are co-transfected with a construct encoding the appropriate MHC I gene into a highly transfectable eukaryotic cell line. The cells transfected with the cDNA pools are then investigated for their ability to stimulate cytokine release from tumour reactive CTL [62]. Single clones are isolated and the peptide epitopes derived from these antigens are identified through the use of HLA peptide-binding motif algorithms [63]. SEREX is a valid alternative molecular approach for TAAs discovery. Unlike molecular approaches that attempt to ascribe immunity to cloned genes, SEREX employs patient sera and autologous tumors to identify TAAs that have elicited high titer IgG antibodies in patients demonstrating immunity to the antigen. This approach has identified a number of gene products that have relevance to tumour development as well as other antigens that may be potential targets for cancer vaccines. Some of the TAAs discovered using SEREX that may be useful, as vaccines include antigens cloned from small cell carcinoma, colon cancer and breast cancer. Another method used in the search for TAAs is *acid elution of peptides from tumour-cell surface MHC I molecules*. Pools of peptides are produced, fractioned with high pressure liquid chromatography, and sequenced [63,64]. The peptides are subsequently tested for their ability to sensitize targets to enable recognition by specific T cells. TAAs that are over-expressed or up-regulated in cancer cells can also be identified by analysis of differentially expressed genes in tumour cells compared to their counterpart normal cells. *Serial analysis of gene expression (SAGE) *is a useful approach to determine the expression of a high number of genes simultaneously. There are many benefits to SAGE, firstly the ability to evaluate the expression pattern of many genes in a quantitative manner without prior sequence information. SAGE can determine the abundance of mRNAs and can also detect small differences in expression levels between samples. The SAGE method is based on two main concepts: *a) *a short sequence tag consisting of 9–10 base pairs (bp) is used to identify a transcript; *b) *serial units of short sequence tags allow the efficient analysis of transcripts by the sequencing of multiple tags with a single clone. Since the introduction of differential display by Liang and Pardee [65], the rate at which differentially expressed genes have been identified has dramatically increased. The abundance of genes is a result of the simplicity and sensitivity of differential display and also the capability of this method to compare multiple RNA samples in order to distinguish between up-regulated and down-regulated genes without knowing their sequences. Differential display employs PCR and DNA gel electrophoresis: compared to other methods, it is more sensitive and only a few picograms of total RNA is needed, and results are highly reproducible from run to run (>90%).

## Cancer-testis antigens: a new class of biomarkers

CT antigens represents a category of TAA normally expressed in male germ cells but not in adult somatic tissues [66]. Since the restricted CT expression profile, the presence of multi-gene family, and the X chromosomal location of all the SEREX identified antigens, has been originally proposed the term of CT antigen to encompass the heterogeneous group of antigens. Although, earlier studies of CT antigens defined common features of these antigens, namely the cancer/testis-restricted expression profile, presence of multi-gene families, mapping to the X chromosome, and immunogenicity in cancer patients, subsequently additional features have been identified. Among them: (*a) *heterogeneous protein expression in cancer; *(b) *correlation of mRNA expression with tumour progression and with tumors of higher malignant potential; *(c) in vitro *activation by hypomethylation and/or histone deacetylation.

Actually, 44 distinct CT "gene" or "antigen" families have been reported in literature. Certain CT gene families contain multiple members, as well as splice variants and today a total of 89 distinct transcripts are know to be encoded by CT genes [67]. By comparing CT antigen expression data the following comments have been made [67]:

• the expression frequency of a single CT antigen is highly variable in different tumors type;

• the frequency of different CT antigen expression in a tumour type can be different;

• there is a tendency for CT antigen expression to be clustered, *i.e*. certain tumour specimens are found to express multiple CT antigens simultaneously, whereas other tumors will be totally negative for CT antigens.

Excluding nervous system and pancreatic cancers, which have been still insufficiently [68,69] studied with regard to CT transcript expression, the tumour types can be distinct in three distinct groups on the basis of the number of CT genes expressed and their expression frequency:

a) *high CT expressors*: tumors which express more than 50% of the CT-antigens;

b) *moderate CT expressors*: tumors which express between 30–50% of the CT transcripts;

c) *low CT expressors*: tumors which express less than 30% of the CT transcripts.

These data are taken from mRNA expression frequencies, which do not necessarily directly correlate with protein expression frequencies. Discrepancies between gene and protein expression frequencies may reflect variations in tissue sampling, tumour heterogeneity or different level of sensitivity of detection. The expression of these antigens may however correlate with advanced stage and grade of tumors, and the presence of spontaneous immune reactivity to these antigens has been shown to correlate with improved prognosis in some studies.

## Cancer-testis antigens expression in HCC

A number of CT antigens have been found expressed with a high percentage and specificity in HCC and their products are promising targets for antigen-specific immunotherapy of this tumor. Moreover, the high frequent co-expression of multiple members of CT antigens in HCC provides possibility of polyvalent vaccinations for HCC.

Zhao *et al *reported that of 105 HCC tissues, MAGE1, SSX-1, CTp11 and HCA587 mRNA expressions were detectable in 75.2%, 72.4%, 62.9% and 56.2% of HCC samples, respectively [70]. About 93.3%, 72.4%, 48.6% and 37.1% of HCC tissues positively expressed at least one, two, three, and four members of CT antigens, respectively. Conversely, only SSX-1 could be detectable in 2.9% of the corresponding adjacent non-HCC tissues in which no metastatic lesion was found. Interestingly, in that study the serum AFP was normal (< 20 ng/mL) or slightly elevated (< 40 ng/mL) in 30.0% of HCC patients. In these patients, 91% had MAGE-1, SSX1, CTp11 or HCA587 transcripts in their HCC tissues, suggesting applying their mRNAs as tumour-specific markers to detect HCC cells in circulation might be an adjuvant diagnostic tool [70].

The expression of the CT antigens mRNA was also investigated by Wu *et al*. in the tissues of HCC and corresponding peripheral blood of 37 patients with HCC [70]. Fifteen samples of cirrhotic tissues and 15 normal tissues were examined with the same method. Two kinds of CT antigens (SSX-2 and SSX-5) showed high-specific and high-frequent expression in HCC tissues, but neither of them could be detected in adjacent non-HCC tissues. In corresponding peripheral blood of HCC tissues, the positive expression rate of the SSX-2 and SSX-5 mRNA was not very high. No relationship was found between the expression of CT antigens and clinical indicators such as age, sex, tumor size, TNM staging, serum AFP level and infection with hepatitis virus [71]. In 15 patients with cirrhosis and 15 other non-tumor patients, none of the SSX-2 and SSX-5 mRNA was detected in liver tissue or peripheral blood. High frequency and specificity of CT antigens in HCC indicates that their products may be new potential promising targets for antigen-specific immunotherapy of HCC. Moreover, the high frequent co-expression of the two genes in HCC provides a possibility of polyvalent vaccinations for this neoplasia. Specific expression of CT antigens was observed in AFP-negative HCC, suggesting the application of their mRNA as tumor markers to detect circulating HCC cells, as adjuvant diagnostic tool, and as indicators of recurrence and prognosis. Peng *et al*. found that the expression of MAGE-A3, SSX-1, SSX-2, SSX-4, MAGE-B2, MAGE-C1, and MAGE-C2 correlated significantly with older age [72]. Moreover, the expressions of MAGE-A4 and SCP-1 were related to AFP abnormality, and the expression of NY-ESO-1 was related to early tumor stage [72]. There was no correlation observed between the expression of CT antigens and the sex, HBV infection or tumor size [72].

In search for genes associated with HCC by cDNA micro-array, Yin *et al*. found that the transcription of TSPY, "testis-specific protein Y-encoded", was up-regulated in HCC, and that TSPY is a novel CT antigen which might be serve as a potential candidate in vaccine strategy for immunotherapy in HCC patients [73].

NY-ESO-1 and LAGE-1 are expressed in a high percentage of HCC, especially in cases with metastasis [74]. It is thus possible that also NY-ESO-1/LAGE-1 might serve as targets for antigen-specific immunotherapy in HCC and NY-ESO-1 peptide vaccination may be of use for patients with advanced HCC [74]. Positive rate of NY-ESO-1 mRNA was 27.4% in HCC; it was higher in HCC with tumor embolus of portal vein than in HCC without tumor embolism (40.0% *versus *18.9%). Positive rate of NY-ESO-1 protein was 18.9% in HCC tissue micro-array; it was significantly higher in HCC with metastasis than in HCC without metastasis (29.6% *versus *11.5%,). Positive rates of NY-ESO-1 mRNA and protein were 28.3% and 19.1% respectively in HBsAg positive HCC, and were 29.5% and 20.7% respectively in HCC with AFP of > 20 ng/ml. Both NY-ESO-1 mRNA and protein were not detected in adjacent normal liver tissue [75].

Recently, Shi *et al*. identified another antigen of great interest called transgelin 2 [76]. The over-expression of transgelin 2 mRNA in a large per cent (69%) of HCC points to its potential as a diagnostic marker for this cancer.

Li *et al *showed that in HCC specimens the HCA587 (the HCA587 gene, identified by SEREX from a HCC patient, encodes a new member of CT antigens) protein was expressed in 37.1% samples. The expressed protein was either detected in the cytoplasm or nucleus depending on the individual samples. More importantly, there appears to be correlation between the tumor differentiation of HCC and HCA587 protein expression, i.e., the lower differentiation, the higher percentage of protein expression [77].

From the data of Luo *et al*. it appears that about >80% of the patients with HCC would be eligible for specific immunotherapeutic approaches with at least one CT antigen in ways similar to those currently being evaluated in malignant melanomas, which express CT antigens at a similar frequency as HCC [78]. While all patients with a tumor expressing a given CT antigen would be candidates for vaccine strategies using whole antigenic proteins, the percentage of patients eligible for peptide-specific vaccination would be much lower, since it requires antigenic peptides with binding motifs restricted to specific MHC alleles.

## Concluding remarks

It is indubitable that there is a growing interest in identifying diagnostic tools that could complement standard histopathology to determine the presence of cancer cells in tissues [79]. In particular, a pressing need exists for biomarkers useful for early cancer detection, accurate pretreatment staging, prediction of response to treatment, and monitoring of disease progression [80].

The availability of a suitable serological marker to distinguish between HCC and benign liver lesions would be very useful for early diagnosis. The only serological marker currently widely used for the diagnosis of HCC is AFP. However, the sensitivity of this marker is limited. In addition, AFP is not always elevated in the early stages of cancer development, when therapy is mostly effective. Given the high heterogeneity of HCC, it is currently thought that an optimal serological test for HCC will be based on the simultaneous measurement of two or three highly specific serological markers [24].

Although several studies have proposed many serum markers, everyone agrees that it is difficult to accurately diagnose early HCC. But early detection is still the only way to improve mortality – the disease is nearly always fatal once the tumors are no longer amenable to surgical or ablative approaches [81] In an effort to find a more reliable serum test for early HCC the National Cancer Institute has launched a 2-years study to validate the usefulness of DCP as a molecular marker [81]. In Japan, where HCV causes 80% of HCC and HBV causes another 10%, have been implemented guidelines describing an ideal screening program for high-risk patients. The program consists of checking the tumor markers AFP and DCP every month, performing abdominal ultrasonography every three months, and performing abdominal computed tomography every 6 months.

There is growing evidence for a link between the immune system and the control of cancer. Emerging evidence indicate, however, that the markers would need to be studied for each tumor type. Since the markers studied to date are not universally prognostic, further details in approaches for studying these markers are warranted.

Although a number of CT antigens have been discovered and it has been suggested that some of them could be helpful in the early detection of HCC, the complexity of human beings leads us to reflect on the need to establish compelling new criteria for validating their real applicability.

Biological complexity can be intuitively appreciated – at least in terms of morphological or behavioral complexity, or the variety of cell types in an organism – but the term itself is notoriously difficult to define [82].

Human beings are complex hierarchical systems consisting of a number of levels of anatomical organization (genes, cells, tissues, organs, apparatuses, and organism) that interrelate differently with each other to form networks of growing complexity [14,82-85].

In order to understand biology at the system level, we need to examine the structure and dynamics of the functions of organisms rather than the characteristics of their constitutive isolated parts [82-85].

Better diagnostic methods are today needed to increase the survival rate in liver cancer patients [86]. Proteomics can be simply defined as the protein expression of the genome; and protein expression can vary depending on the biological state. Antibody microarrays can scan for multiple targets (antigens) within the tissue or in the circulation. This technology is still in its infancy and has great potential as a diagnostic tool for hepatitis liver cancer patients [87]. Another proteomic approach is mass spectrometry, which can detect proteins and present them as charged ions. The mass spectrometric technique termed *surface enhanced laser desorption ionization *(SELDI), releases proteins in a sample from a capturing surface that can specifically bind groups of proteins which share common features (i.e. hydrophobic, negatively charged) and the expression of thousands of proteins can be monitored simultaneously [87]. Interestingly, Lee *et al*. using SELDI found that complement C3a was elevated in patients with chronic hepatitis C and HCV-related HCC [88].

In the future, gene expression profiling ("disease fingerprinting") is likely to complement liver biopsy in the molecular differential diagnosis of chronic liver diseases and HCC. There has already been some success in the identification of subtypes of HCC based on derived sets of signature gene clusters [10,89].

The expression of CT in many tumoral tissues has mainly been studied at the level of gene expression and gene level measurement by RT-PCR analysis and the quantitative real-time PCR (qrt-PCR) technology [90,91]. However, the information provided by these approaches is limited by the fact that the phenomena observed at each level of anatomical organization have properties that do not exist at a lower or higher level: RT-PCR and qrt-PCR may offer a satisfactory qualitative/quantitative description of small-scale structures, but this is likely to be irrelevant when it comes to large-scale features.

The above considerations, in conjunction with the complexity of tumor-host interactions within the tumor microenvironment caused by temporal changes in tumor phenotypes and an array of immune mediators expressed in the tumor microenvironment might clarify the limited reliability and applicability of current molecular biomarkers.

Despite the rapid advances that have been made in the fields of molecular and cellular biology, there is no doubt that cancer is still a very complex disease: it can be hypothesized that each tumour is unique, and that the spectrum of biological changes determining human tumors is infinitely variable [14,85]. Carcinogenesis is a non-linear process, whose behavior does not follow clearly predictable and repeatable pathways. Periods of inactivity may be punctuated by sudden change, apparent patterns of behavior may disappear and new patterns surprisingly emerge [14,85]. Cancer is determined by a number of processes and controls operating over much broader scales, and by factors such as structural controls that may operate at scales ranging from molecular to environmental. This *multiple scale causality *not only recognizes multiple processes and controls acting at multiple scales but, unlike a strict reductionist approach, may also recognize the fact that relevant "first principles" may reside at scales other than the smallest micro-scales. In other words, the observed phenomenon at each scale has structural and behavioral properties that do not exist at lower or higher organizational levels [14,85].

Viewing cancer as a system that is dynamically complex in time and space will probably reveal more about its underlying behavioral characteristics. This way of thinking may further help to clarify concepts, interpret new and old experimental data, indicate alternative experiments and categorize the acquired knowledge on the basis of the similitude and/or shared behaviors of very different tumors. It is encouraging that mathematics, theoretics, biology and medicine continue to contribute together towards a common quantitative understanding of cancer complexity.

To advance our knowledge in a currently widely debated field of investigation such as that of biomarkers in HCC, a clearer distinction must be made between *in vitro *laboratory results (the discovery and validation of possible biomarkers) and their real application (*in vivo *validation)in human beings, and it is necessary to adopt a more complete experimental approach that forcefully includes both morphological and molecular techniques. Finally, the analysis of these markers could be useful in identifying patients that are most likely to benefit from immune intervention strategies.

## References

[B1] Zerbini A, Pilli M, Ferrari C, Missale G (2006). Is there a role for immunotherapy in hepatocellular carcinoma?. Dig Liver Dis.

[B2] Marrero JA (2006). Hepatocellular carcinoma. Curr Opin Gastroenterol.

[B3] Motola-Kuba D, Zamora-Valdes D, Uribe M, Mendez-Sanchez N (2006). Hepatocellular carcinoma. An overview. Ann Hepatol.

[B4] Jemal A, Murray T, Ward E, Samuels A, Tiwari RC, Ghafoor A, Feuer EJ, Thun MJ (2005). Cancer statistics, 2005. CA Cancer J Clin.

[B5] Jaskiewicz K, Stepien A, Banach L (1991). Hepatocellular carcinoma in a rural population at risk. Anticancer Res.

[B6] Blum HE (2005). Hepatocellular carcinoma: therapy and prevention. World J Gastroenterol.

[B7] Farazi PA, DePinho RA (2006). Hepatocellular carcinoma pathogenesis: from genes to environment. Nat Rev Cancer.

[B8] Kim Y, Sills RC, Houle CD (2005). Overview of the molecular biology of hepatocellular neoplasms and hepatoblastomas of the mouse liver. Toxicol Pathol.

[B9] Cha C, Dematteo RP (2005). Molecular mechanisms in hepatocellular carcinoma development. Best Pract Res Clin Gastroenterol.

[B10] Thorgeirsson SS, Lee JS, Grisham JW (2006). Functional genomics of hepatocellular carcinoma. Hepatology.

[B11] Llovet JM, Burroughs A, Bruix J (2003). Hepatocellular carcinoma. Lancet.

[B12] Kojiro M (2005). Histopathology of liver cancers. Best Pract Res Clin Gastroenterol.

[B13] Grizzi F, Russo C, Portinaro N, Hermonat PL, Chiriva-Internati M (2004). Complexity and cancer. Gastroenterology.

[B14] Grizzi F, Chiriva-Internati M (2006). Cancer: looking for simplicity and finding complexity. Cancer Cell Int.

[B15] Lee JS, Thorgeirsson SS (2004). Genome-scale profiling of gene expression in hepatocellular carcinoma: classification, survival prediction, and identification of therapeutic targets. Gastroenterology.

[B16] Thorgeirsson SS, Grisham JW (2002). Molecular pathogenesis of human hepatocellular carcinoma. Nat Genet.

[B17] Zhou L, Liu J, Luo F (2006). Serum tumor markers for detection of hepatocellular carcinoma. World J Gastroenterol.

[B18] Terentiev AA, Moldogazieva NT (2006). Structural and functional mapping of alpha-fetoprotein. Biochemistry (Mosc).

[B19] Canick JA, MacRae AR (2005). Second trimester serum markers. Semin Perinatol.

[B20] Ding X, Yang LY, Huang GW, Yang JQ, Liu HL, Wang W, Peng JX, Yang JQ, Tao YM, Chang ZG, Ling XS (2005). Role of AFP mRNA expression in peripheral blood as a predictor for postsurgical recurrence of hepatocellular carcinoma: a systematic review and meta-analysis. World J Gastroenterol.

[B21] Abelev GI (1968). Production of embryonal serum alpha-globulin by hepatomas: review of experimental and clinical data. Cancer Res.

[B22] Daniele B, Bencivenga A, Megna AS, Tinessa V (2004). Alpha-fetoprotein and ultrasonography screening for hepatocellular carcinoma. Gastroenterology.

[B23] Yuen MF, Lai CL (2005). Serological markers of liver cancer. Best Pract Res Clin Gastroenterol.

[B24] Gebo KA, Chander G, Jenckes MW, Ghanem KG, Herlong HF, Torbenson MS, El-Kamary SS, Bass EB (2002). Screening tests for hepatocellular carcinoma in patients with chronic hepatitis C: a systematic review. Hepatology.

[B25] Capurro M, Wanless IR, Sherman M, Deboer G, Shi W, Miyoshi E, Filmus J (2003). Glypican-3: a novel serum and histochemical marker for hepatocellular carcinoma. Gastroenterology.

[B26] Filmus J, Capurro M (2004). Glypican-3 and alphafetoprotein as diagnostic tests for hepatocellular carcinoma. Mol Diagn.

[B27] Nakatsura T, Nishimura Y (2005). Usefulness of the novel oncofetal antigen glypican-3 for diagnosis of hepatocellular carcinoma and melanoma. BioDrugs.

[B28] Daubeuf S, Accaoui MJ, Pettersen I, Huseby NE, Visvikis A, Galteau MM (2001). Differential regulation of gamma-glutamyltransferase mRNAs in four human tumour cell lines. Biochim Biophys Acta.

[B29] Cui R, He J, Zhang F, Wang B, Ding H, Shen H, Li Y, Chen X (2003). Diagnostic value of protein induced by vitamin K absence (PIVKAII) and hepatoma-specific band of serum gamma-glutamyl transferase (GGTII) as hepatocellular carcinoma markers complementary to alpha-fetoprotein. Br J Cancer.

[B30] Haydon GH, Hayes PC (1996). Screening for hepatocellular carcinoma. Eur J Gastroenterol Hepatol.

[B31] Li C, Qian J, Lin JS (2006). Purification and characterization of alpha-L-fucosidase from human primary hepatocarcinoma tissue. World J Gastroenterol.

[B32] Giardina MG, Matarazzo M, Varriale A, Morante R, Napoli A, Martino R (1992). Serum alpha L-fucosidase. A useful marker in the diagnosis of hepatocellular carcinoma. Cancer.

[B33] Deugnier Y, David V, Brissot P, Mabo P, Delamaire D, Messner M, Bourel M, Legall JY (1984). Serum alpha-L-fucosidase: a new marker for the diagnosis of primary hepatic carcinoma?. Hepatology.

[B34] Ayude D, Fernandez-Rodriguez J, Rodriguez-Berrocal FJ, Martinez-Zorzano VS, de Carlos A, Gil E, Paez de La Cadena M (2000). Value of the serum alpha-L-fucosidase activity in the diagnosis of colorectal cancer. Oncology.

[B35] Abdel-Aleem H, Ahmed A, Sabra AM, Zakhari M, Soliman M, Hamed H (1996). Serum alpha L-fucosidase enzyme activity in ovarian and other female genital tract tumors. Int J Gynaecol Obstet.

[B36] Marrero JA, Su GL, Wei W, Emick D, Conjeevaram HS, Fontana RJ, Lok AS (2003). Des-gamma carboxyprothrombin can differentiate hepatocellular carcinoma from nonmalignant chronic liver disease in american patients. Hepatology.

[B37] Suzuki M, Shiraha H, Fujikawa T, Takaoka N, Ueda N, Nakanishi Y, Koike K, Takaki A, Shiratori Y (2005). Des-gamma-carboxy prothrombin is a potential autologous growth factor for hepatocellular carcinoma. J Biol Chem.

[B38] Semela D, Dufour JF (2004). Angiogenesis and hepatocellular carcinoma. J Hepatol.

[B39] Moon WS, Rhyu KH, Kang MJ, Lee DG, Yu HC, Yeum JH, Koh GY, Tarnawski AS (2003). Overexpression of VEGF and angiopoietin 2: a key to high vascularity of hepatocellular carcinoma?. Mod Pathol.

[B40] Huang GW, Yang LY, Lu WQ (2005). Expression of hypoxia-inducible factor 1alpha and vascular endothelial growth factor in hepatocellular carcinoma: Impact on neovascularization and survival. World J Gastroenterol.

[B41] Arii S (2004). Role of vascular endothelial growth factor on the invasive potential of hepatocellular carcinoma. J Hepatol.

[B42] Kanematsu M, Osada S, Amaoka N, Goshima S, Kondo H, Kato H, Nishibori H, Yokoyama R, Hoshi H, Moriyama N (2005). Expression of vascular endothelial growth factor in hepatocellular carcinoma and the surrounding liver and correlation with MRI findings. AJR Am J Roentgenol.

[B43] Kubo F, Ueno S, Hiwatashi K, Sakoda M, Kawaida K, Nuruki K, Aikou T (2005). Interleukin 8 in human hepatocellular carcinoma correlates with cancer cell invasion of vessels but not with tumor angiogenesis. Ann Surg Oncol.

[B44] Ren Y, Poon RT, Tsui HT, Chen WH, Li Z, Lau C, Yu WC, Fan ST (2003). Interleukin-8 serum levels in patients with hepatocellular carcinoma: correlations with clinicopathological features and prognosis. Clin Cancer Res.

[B45] Takeda A, Stoeltzing O, Ahmad SA, Reinmuth N, Liu W, Parikh A, Fan F, Akagi M, Ellis LM (2002). Role of angiogenesis in the development and growth of liver metastasis. Ann Surg Oncol.

[B46] Song BC, Chung YH, Kim JA, Choi WB, Suh DD, Pyo SI, Shin JW, Lee HC, Lee YS, Suh DJ (2002). Transforming growth factor-beta1 as a useful serologic marker of small hepatocellular carcinoma. Cancer.

[B47] Schulte-Hermann R, Bursch W, Low-Baselli A, Wagner A, Grasl-Kraupp B (1997). Apoptosis in the liver and its role in hepatocarcinogenesis. Cell Biol Toxicol.

[B48] Liang YR, Wan DS, Chen G, Lu ZH, Li YJ, Lin YH, Chi PD (2002). Detection of serum tumor supplied group of factors and its clinical significance. Ai Zheng.

[B49] Ding X, Yang LY, Huang GW, Yang JQ, Liu HL, Wang W, Peng JX, Yang JQ, Tao YM, Chang ZG, Ling XS (2005). Role of AFP mRNA expression in peripheral blood as a predictor for postsurgical recurrence of hepatocellular carcinoma: a systematic review and meta-analysis. World J Gastroenterol.

[B50] Matsumura M, Niwa Y, Kato N, Komatsu Y, Shiina S, Kawabe T, Kawase T, Toyoshima H, Ihori M, Shiratori Y (1994). Detection of alpha-fetoprotein mRNA, an indicator of hematogenous spreading hepatocellular carcinoma, in the circulation: a possible predictor of metastatic hepatocellular carcinoma. Hepatology.

[B51] Sheen IS, Jeng KS, Tsai YC (2003). Is the expression of gamma-glutamyl transpeptidase messenger RNA an indicator of biological behavior in recurrent hepatocellular carcinoma?. World J Gastroenterol.

[B52] Pettersen I, Andersen JH, Bjornland K, Mathisen O, Bremnes R, Wellman M, Visvikis A, Huseby NE (2003). Heterogeneity in gamma-glutamyltransferase mRNA expression and glycan structures. Search for tumor-specific variants in human liver metastases and colon carcinoma cells. Biochim Biophys Acta.

[B53] Chen CJ, Kyo S, Liu YC, Cheng YL, Hsieh CB, Chan DC, Yu JC, Harn HJ (2004). Modulation of human telomerase reverse transcriptase in hepatocellular carcinoma. World J Gastroenterol.

[B54] Onishi T, Nouso K, Higashi T, Toshikuni N, Nakatsukasa H, Kobayashi Y, Uemura M, Yumoto E, Fujiwara K, Sato S, Nakamura S, Yokoyama J, Hanafusa T, Shiratori Y (2003). Cellular distribution of telomerase reverse transcriptase in human hepatocellular carcinoma. J Gastroenterol Hepatol.

[B55] Fu XM, Yang QX, Shao CK, Feng ZY (2006). Expressions of h-TERT, c-myc, PCNA and cell apoptosis in liver carcinogenesis. Nan Fang Yi Ke Da Xue Xue Bao.

[B56] Oh BK, Kim YJ, Park YN, Choi J, Kim KS, Park C (2005). Quantitative assessment of hTERT mRNA expression in dysplastic nodules of HBV-related hepatocarcinogenesis. World J Gastroenterol.

[B57] Himoto T, Kuriyama S, Zhang JY, Chan EK, Kimura Y, Masaki T, Uchida N, Nishioka M, Tan EM (2005). Analyses of autoantibodies against tumor-associated antigens in patients with hepatocellular carcinoma. Int J Oncol.

[B58] Parish CR (2003). Cancer immunotherapy: the past, the present and the future. Immunol Cell Biol.

[B59] Burnet FM (1967). Immunological aspects of malignant disease. Lancet.

[B60] Lewis JD, Reilly BD, Bright RK (2003). Tumor-associated antigens: from discovery to immunity. Int Rev Immunol.

[B61] van der Bruggen P, Traversari C, Chomez P, Lurquin C, De Plaen E, Van den Eynde B, Knuth A, Boon T (1991). A gene encoding an antigen recognized by cytolytic T lymphocytes on a human melanoma. Science.

[B62] Rosenberg SA (2000). Principles and Practice of the Biologic Therapy of Cancer.

[B63] Parker KC, Shields M, DiBrino M, Brooks A, Coligan JE (1995). Peptide binding to MHC class I molecules: implications for antigenic peptide prediction. Immunol Res.

[B64] van Bleek G, Nathenson S (1990). Isolation of an endogenously processed immunodominant viral peptide from the class I H-2Kb molecule. Nature.

[B65] Liang P, Pardee AB (1992). Differential display of eukaryotic messenger RNA by means of the polymerase chain reaction. Science.

[B66] Scanlan MJ, Gure AO, Jungbluth AA, Old LJ, Chen YT (2002). Cancer/testis antigens: an expanding family of targets for cancer immunotherapy. Immun Rev.

[B67] Scanlan MJ, Simpson AJG, Old LJ (2004). The cancer/testis genes: review, standardization, and commentary. Cancer Immunity.

[B68] Grizzi F, Gaetani P, Franceschini B, Di Ieva A, Colombo P, Ceva-Grimaldi G, Bollati A, Frezza EE, Cobos E, Rodriguez y, Baena R, Dioguardi N, Chiriva-Internati M (2006). Sperm protein 17 is expressed in human nervous system tumours. BMC Cancer.

[B69] Kubuschok B, Xie X, Jesnowski R, Preuss KD, Romeike BF, Neumann F, Regitz E, Pistorius G, Schilling M, Scheunemann P, Izbicki JR, Lohr JM, Pfreundschuh M (2004). Expression of cancer testis antigens in pancreatic carcinoma cell lines, pancreatic adenocarcinoma and chronic pancreatitis. Int J Cancer.

[B70] Zhao L, Mou DC, Leng XS, Peng JR, Wang WX, Huang L, Li S, Zhu JY (2004). Expression of cancer-testis antigens in hepatocellular carcinoma. World J Gastroenterol.

[B71] Wu LQ, Lu Y, Wang XF, Lv ZH, Zhang B, Yang JY (2006). Expression of cancer-testis antigen (CTA) in tumor tissues and peripheral blood of Chinese patients with hepatocellular carcinoma. Life Sci.

[B72] Peng JR, Chen HS, Mou DC, Cao J, Cong X, Qin LL, Wei L, Leng XS, Wang Y, Chen WF (2005). Expression of cancer/testis (CT) antigens in Chinese hepatocellular carcinoma and its correlation with clinical parameters. Cancer Lett.

[B73] Yin YH, Li YY, Qiao H, Wang HC, Yang XA, Zhang HG, Pang XW, Zhang Y, Chen WF (2005). TSPY is a cancer testis antigen expressed in human hepatocellular carcinoma. Br J Cancer.

[B74] Zhang WM, Xiao G, Zhang M, Guo AL, Dong Y, Wen JM (2005). Expression of NY-ESO-1 and LAGE-1 cancer-testis antigens in hepatocellular carcinoma. Zhonghua Bing Li Xue Za Zhi.

[B75] Zhang WM, Xiao G, Xie D, Zhang M, Guo AL, Wen JM (2005). Correlation of NY-ESO-1 gene and protein expression to metastasis and clinicopathologic features of hepatocellular carcinoma. Ai Zheng.

[B76] Shi YY, Wang HC, Yin YH, Sun WS, Li Y, Zhang CQ, Wang Y, Wang S, Chen WF (2005). Identification and analysis of tumour-associated antigens in hepatocellular carcinoma. Br J Cancer.

[B77] Li B, Qian XP, Pang XW, Zou WZ, Wang YP, Wu HY, Chen WF (2003). HCA587 antigen expression in normal tissues and cancers: correlation with tumor differentiation in hepatocellular carcinoma. Lab Invest.

[B78] Luo G, Huang S, Xie X, Stockert E, Chen YT, Kubuschok B, Pfreundschuh M (2002). Expression of cancer-testis genes in human hepatocellular carcinomas. Cancer Immun.

[B79] Bast RC, Lilja H, Urban N, Rimm DL, Fritsche H, Gray J, Veltri R, Klee G, Allen A, Kim N, Gutman S, Rubin MA, Hruszkewycz A (2005). Translational crossroads for biomarkers. Clin Cancer Res.

[B80] Basil CF, Zhao Y, Zavaglia K, Jin P, Panelli MC, Voiculescu S, Mandruzzato S, Lee HM, Seliger B, Freedman RS, Taylor PR, Hu N, Zanovello P, Marincola FM, Wang E (2006). Common cancer biomarkers. Cancer Res.

[B81] Wilson JF (2005). Liver cancer on the rise. Ann Intern Med.

[B82] Grizzi F, Chiriva-Internati M (2005). The complexity of anatomical systems. Theor Biol Med Model.

[B83] Szathmary E, Jordan F, Pal C (2001). Molecular biology and evolution. Can genes explain biological complexity?. Science.

[B84] Nurse P (1997). Reductionism. The ends of understanding. Nature.

[B85] Grizzi F, Di Ieva A, Russo C, Frezza EE, Cobos E, Muzzio PC, Chiriva-Internati M (2006). Cancer initiation and progression: an unsimplifiable complexity. Theor Biol Med Model.

[B86] Spangenberg HC, Thimme R, Blum HE (2006). Serum markers of hepatocellular carcinoma. Semin Liver Dis.

[B87] El-Aneed A, Banoub J (2006). Proteomics in the diagnosis of hepatocellular carcinoma: focus on high risk hepatitis B and C patients. Anticancer Res.

[B88] Lee IN, Chen CH, Sheu JC, Lee HS, Huang GT, Chen DS, Yu CY, Wen CL, Lu FJ, Chow LP (2006). Identification of complement C3a as a candidate biomarker in human chronic hepatitis C and HCV-related hepatocellular carcinoma using a proteomics approach. Proteomics.

[B89] Lemmer ER, Friedman SL, Llovet JM (2006). Molecular diagnosis of chronic liver disease and hepatocellular carcinoma: the potential of gene expression profiling. Semin Liver Dis.

[B90] Chiriva-Internati M, Grizzi F, Bright RK, Martin Kast W (2004). Cancer immunotherapy: avoiding the road to perdition. J Transl Med.

[B91] Juretic A, Spagnoli GC, Schultz-Thater E, Sarcevic B (2003). Cancer/testis tumour-associated antigens: immunohistochemical detection with monoclonal antibodies. Lancet Oncol.

